# Review of *Afraustraloderes rassei* Bouyer, 2012: description of its female and a new species of *Pixodarus* Fairmaire, 1887 (Coleoptera, Cerambycidae, Prioninae)

**DOI:** 10.3897/zookeys.558.6112

**Published:** 2016-02-01

**Authors:** Anders Bjørnstad, Elizabeth Grobbelaar, Renzo Perissinotto

**Affiliations:** 1 Høyåsstien 12, N-3727 Skien, Norway; 2 South African National Collection of Insects, Plant Protection Research Institute, Agricultural Research Council, Private Bag X134 Queenswood, 0121 Pretoria, South Africa; 3 DST/NRF Research Chair, Nelson Mandela Metropolitan University, P.O. Box 77000, Port Elizabeth 6031, South Africa

**Keywords:** Hopliderini, new species, Cape Floral Region, South Africa

## Abstract

The original description of *Afraustraloderes rassei* Bouyer, 2012 included a female that is now recognized as a separate species belonging to the genus *Pixodarus* and here described as *Pixodarus spiniscapus* sp. n. The true female of *Afraustraloderes rassei* has also been obtained recently and is, therefore, here described. The synonymy of *Pixodarus exasperatus* with *Pixodarus nyassae*, proposed earlier by Santos Ferreira (1980), is here supported. Conversely, the earlier inclusion of *Afraustraloderes rassei* in the tribe Hopliderini is rejected, on the basis of a key set of characters established by Quentin and Villiers (1972, 1975). *Afraustraloderes rassei* appears to be restricted to the Cape Floral Region, exhibiting larval development in trunks and roots of dead Proteaceae plants. Conversely, *Pixodarus spiniscapus* has so far only been recorded in the eastern part of South Africa and appears to be associated with bushveld vegetation.

## Introduction

*Afraustraloderes rassei* Bouyer, 2012 was originally described based on a small collection of three males and one female from South Africa. Bouyer (2012) acknowledged that the two sexes were morphologically very different, but decided nevertheless to describe them as the male and female of a new species and new genus. This was on the grounds that both exhibited a pronounced spine on the internal margin of the first antennomere, which is a unique character not shared with any other known Hopliderini, the tribe to which he assigned the new species.

**Figure 1.**
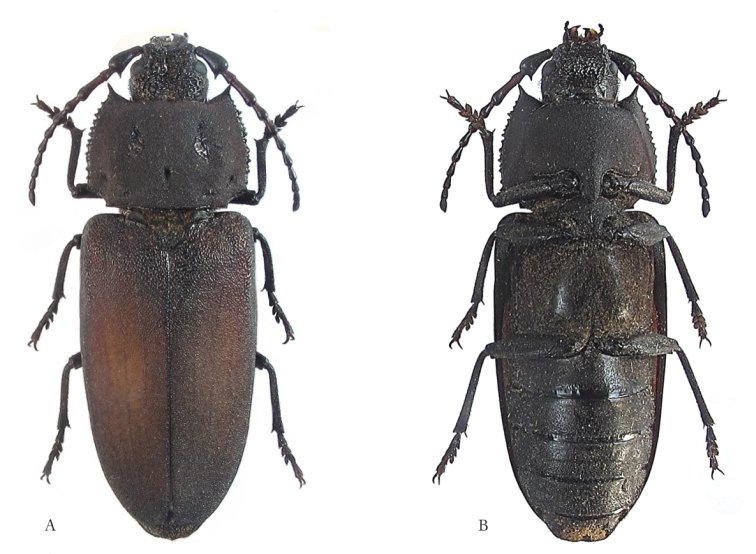
*Afraustraloderes rassei* ♂, 33 mm **A** dorsal habitus **B** ventral habitus (Photos: Lynette Clennell).

The male and female specimens represented in the photos of Figures 1 and 2 in Bouyer (2012), show several distinct differences. The holotype male was purchased at an unspecified entomological fair, and was collected in “Matrosberg” [sic], probably Matroosberg in the Western Cape Province, in 2003. Two paratype males were included in the original description, both from Joubertina in the Eastern Cape Province. The “allotype female”, however, is a specimen reportedly collected in Oribi Gorge, KwaZulu-Natal Province in 1989.

**Figure 2.**
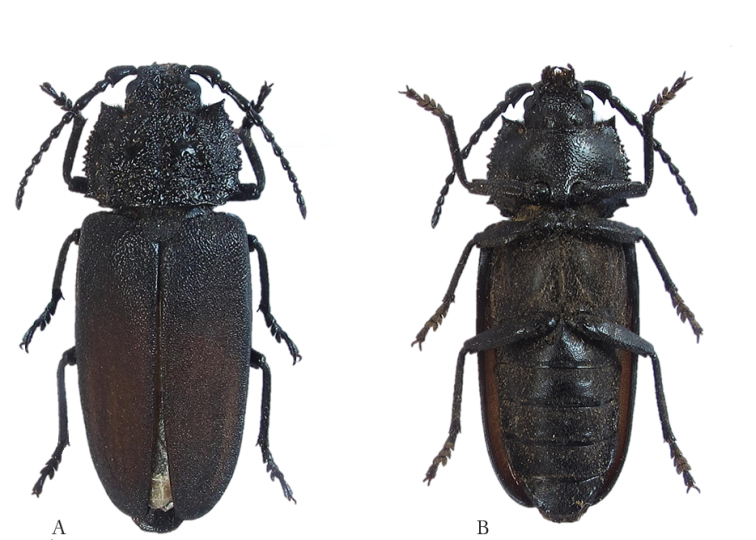
*Afraustraloderes rassei* ♀, 31.5 mm **A** dorsal habitus **B** ventral habitus (Photos: Lynette Clennell).

In 2006, one of the authors (EG) was working on the collection of the late Richard Watmough, a well-known South African entomologist who passed away in 2005 (Allsopp 2005). Upon his death, his private insect collection was bequeathed to the Mountain Club of South Africa, which in turn donated it to the South African National Collection of Insects (SANC), Pretoria. Three specimens resembling the female “*Afraustraloderes rassei* allotype” of Bouyer (2012) were discovered in this collection during 2013, two males and one female. This revealed that the initial assumption that the female belonged to the same species (*Afraustraloderes rassei*) was incorrect, and that this specimen, and those discovered in the Watmough collection, represented a new prionine species. This prompted further searches in the Joubertina fynbos area, in an attempt to find the yet undescribed female of *Afraustraloderes rassei*. One adult female and five more males were obtained in December 2014, by rearing larvae collected in the field during 2013. This now allows for a full review of the species, including the description of the correctly recognised female *Afraustraloderes rassei* and the placement of the original “female allotype” within a new species of *Pixodarus* Fairmaire, 1887.

## Materials and methods

Adult specimens of *Afraustraloderes rassei* were obtained either through direct searching of burnt and dead logs/roots of Proteaceae in the fynbos vegetation or by rearing their larvae in a controlled, closed environmental room in Port Elizabeth, about 170 km from the Joubertina collection locality. Larvae were collected in an advanced stage of development and maintained in the logs in which they were found in closed plastic crates, in darkness and at ambient temperature. Logs were left lying in a horizontal position with minimal manipulation and moistened by spraying rainwater on the outer surface once a month.

Specimen length was measured from the anterior margin of the head to the elytral apex and the width was measured at the widest point of the elytra. Photos of set specimens were taken using either a Canon EOS 5D camera fitted with a Canon MP-E 65 Macro 2.8–1.5× objective or a Canon PowerShot G11 with automatic macro setting. The background was removed from the photos using Microsoft Word 2010 (Picture Tools), to increase clarity of resolution. Combine ZP Image Stacking Software by Alan Hadley (alan@micropics.org.uk) was used to obtain z-stacking composite images. *In situ* photos were taken using a Ricoh CX1 camera with macro setting.

Terminology used to describe the male genitalia follows the standard work of Ehara (1954) and is complemented with more recent views as reported in Hubweber and Schmitt (2006, 2010) and Lin and Li (2012).

Holotype label data is quoted verbatim. Collections are abbreviated as follows: SANC, South African National Collection of Insects (Pretoria, South Africa); NHMO, Natural History Museum (Oslo, Norway); ABPC, Anders Bjørnstad Private Collection (Skien, Norway); RPPC, Renzo Perissinotto and Lynette Clennell Private Collection (Port Elizabeth, South Africa); TBPC, Thierry Bouyer Private Collection (Chênée, Belgium); NDPC, Norbert Delahaye Private Collection (Plaisir, France); TGPC, Thierry Garnier Private Collection (Montpellier, France). Geographical abbreviations are as follows: RSA, Republic of South Africa; ECA, Eastern Cape Province, RSA; GAU, Gauteng Province, RSA; MPU, Mpumalanga province, RSA.

Four observations of the new *Pixodarus* species were located on the citizen science platform iSpot (Silvertown et al. 2015) and incorporated in this study. Stable, permanent copies, using WebCite (Eysenbach and Trudel 2005), were created on 03 July 2015. Their URLs are given in the Additional records section for this species.

## Taxonomic account

### Genus *Afraustraloderes* Bouyer, 2012

**Type species.** *Afraustraloderes rassei* Bouyer, 2012

When Bouyer (2012) described the genus *Afraustraloderes* he assigned it to the tribe Hopliderini Thomson, 1864. The reason for this seems to be due to the incorrect assumption that it was the male of a female which obviously belongs to the Hopliderini.

The tribe Hopliderini was revised by Quentin and Villiers (1972, 1975). They provided several morphological characteristics for recognizing the group, first as a subtribe of the Callipogonini Thomson, 1861 (Quentin and Villiers 1972), later as a tribe of its own (Quentin and Villiers 1975). The most important of these are listed below and numbered here for easy reference:

1. eyes strongly emarginate, but not embracing antennal tubercle;
2. eyes widely separated dorsally;
3. antennae as long as or exceeding the body length in males;
4. antennae gradually becoming more slender distally;
5. scape very short;
6. 3rd antennomere very long;
7. anterior coxal cavities open posteriorly;
8. prosternal process pointed;
9. metathoracic episterna usually truncate apically, without posterior constriction;
10. tibiae apically spined;
11. pronotal disc distinctly punctate;
12. lateral margins of pronotum each bearing five spines;
13. anterior border of pronotum with a silky tuft on each side of the ‘neck’;
14. head strongly declivous, resulting in mandibles not being visible in dorsal view.

*Afraustraloderes* has some of these characteristics: *viz.* items 1, 2, 5, 7, 13 (partly) and 14. But it does not exhibit the majority of the features listed above: *viz.* nos. 3, 4, 6, 8, 9, 10, 11, 12. Regarding no. 9, although the metathoracic episterna are truncate apically, there is an evident constriction near the distal end. Regarding no. 10, there are the usual two spurs between the end of the tibia and the base of the tarsus, but there are no apical teeth or spines on the tibiae themselves. Regarding no. 11, there is micro-punctation on the disc of the male, but not in the female. Thus, from the above analysis it can be concluded that *Afraustraloderes* does not belong in the tribe Hopliderini. We propose that this genus be removed from the Hopliderini and regarded as Prioninae *incertae sedis*.

### Afraustraloderes rassei

Taxon classificationAnimaliaColeopteraCerambycidae

Bouyer, 2012

**Figure 3.**
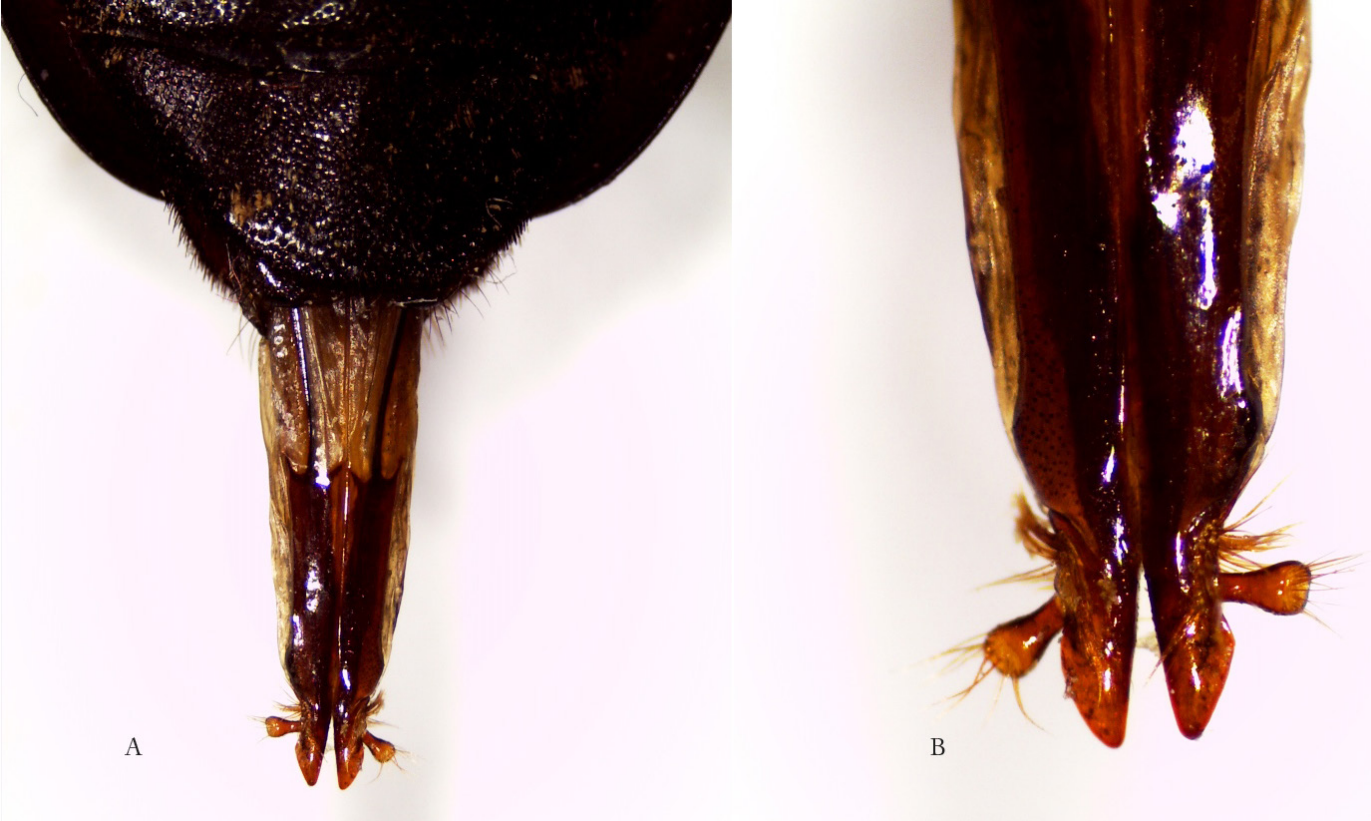
*Afraustraloderes rassei* ♀ **A** ovipositor in dorsal view (length segment 8+9: 6 mm) **B** apical section of ovipositor enlarged (Photos: Anders Bjørnstad).

**Figure 4.**
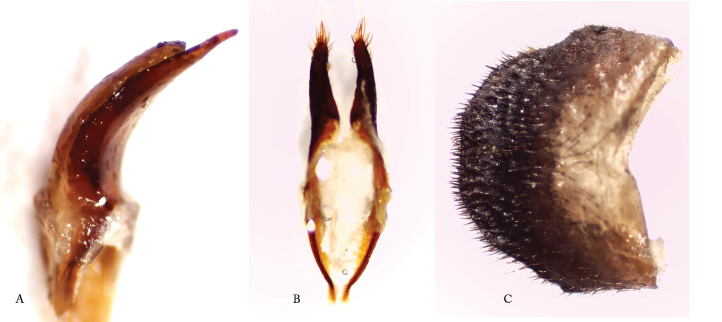
*Afraustraloderes rassei*, male genitalia **A** median lobe, apex of ventral (above) + dorsal (below) plates, semi-lateral view **B** tegmen, ventral view **C** anal tergite, dorsal view (Photos: Anders Bjørnstad).

Figures 1 , 2 , 3 , 4 , 7 , 8

Afraustraloderes rassei Bouyer, 2012: 214

#### Material examined.

Paratypes: 1♂ RSA, ECA, Joubertina (33°53'10” S, 23°50'18” E), 18 Dec 2009, M Villet and R Smith leg (RPPC); 1♂, same locality, but 25 Dec 2010, R Perissinotto and L Clennell (TBPC). Other material: 1♂, same locality as paratype, but Oct 2010, found dead on the ground, Rodger Smith leg (RPPC); 1♀, same locality, but 28 Dec 2014, found dead inside dead *Protea* log, R Perissinotto and L Clennell (RPPC); 5♂ 1♀, same locality, bur reared from larvae in Port Elizabeth, adults emerged 23 Dec 2014-7 Jan 2015 (ABPC, NDPC, NHMO, RPPC, SANC, TGPC).

#### Description of the female.

*Size*. 29.6–31.5 mm long including pygidium, 11.5–12.2 mm wide (maximum width at metacoxae).

*Head*. Mandibles relatively short and broad with foveolate base, distal part short, shiny and weakly arcuate with pointed apex, cutting edge with a small tooth near base; maxillary and labial palpi with terminal segments terete and slightly truncate at apex; galeae much shorter than palpi and covered in rufous bristles; clypeus with long stiff rusty brown bristles directed anteriorly; frons very uneven; antennal tubercles moderately raised, with strongly uneven, deeply sculptured surface; eyes small, finely facetted with deep emargination dividing each eye into subequal lobes, with lower lobe almost reaching gula; vertex strongly uneven and deeply sculpted, with poorly defined median depression.

*Antenna*. Short and slender, only reaching slightly beyond humeri of elytra; with scape as longest antennomere, finely punctate with a narrow base gradually widening distally and ending in apical spine on posterior margin; antennomeres 2–10 with circular cross-section, exhibiting narrow base but widened apically; 11^th^ antennomere slightly compressed; pedicel very short, 3^rd^ antennomere almost as long as scape, antennomeres 4–11 subequal in length.

*Pronotum*. Distinctly transverse, with disc deeply sculpted by irregular reticulations and foveolations; two strongly uneven, raised areas with smooth and shiny surface present about halfway between anterior and posterior margin; anterior margin with fringe of short, black setae curled around base of head, but slightly longer and straight on either side of head, without forming a marked ‘brush’; lateral margins with many (15–20) short teeth, foremost tooth much stronger than others and directed forwards, last tooth on each side, near posterior corner, much larger and sharper than the rest.

*Scutellum*. Broadly tongue-shaped to triangular, with finely reticulate surface.

*Elytron*. Shallowly reticulate basally, sculpture becoming less distinct towards rounded apices; humeri smoothly rounded.

*Legs*. Short and slender; femora only slightly thickened at middle, hardly projecting beyond lateral borders of elytra; tibiae nearly straight, only slightly curved in basal part; tarsi with first tarsomere slightly longer than others, noticeably so in metatarsi.

*Pygidium*. Tergite 8 long, protruding beyond elytral apices.

*Ventral surface*. Base of maxilla punctate and shiny; submentum transversely ridged; gula glabrous, deeply punctate/reticulate/foveolate, but posterior part raised, exhibiting black bristles and less sculpture; prosternum shiny, transversally strongly convex, punctate, glabrous and with ligulate prosternal process bent dorsally at apex; proepimeron only weakly punctate, glabrous, not reaching prosternal process (i.e. procoxal cavities open); mesosternum punctate and with longitudinal median furrow, with soft black pubescence, exhibiting mesial depression just in front of mesocoxae; mesosternal process short, strongly excavate, resulting in bifurcate apex; mesocoxae moderately raised; metasternum transversally strongly convex, with median furrow increasing in depth posteriorly; whole metasternum and mesepisterna covered by soft, sparse blackish pubescence; metathoracic episterna posteriorly narrowed and truncate; all five visible abdominal sternites subequal in length, densely, but shallowly punctate, glabrous in median part, increasingly pubescent laterally with very short and soft whitish bristles; last visible sternite with evenly rounded posterior margin.

*Ovipositor*. Abdominal segments 8 + 9 (Figure 3A) are fully extended, and together measure 6 mm. They telescope into segment 7 when retracted (Hutcheson 1980). Figure 3B shows an enlarged photo of the apical portion of the ovipositor with coxites and styli. The strong sclerotization is consistent with an ovipositor characteristic of Cerambycidae that lay their eggs beneath bark, and is typical of the subfamily Prioninae, as opposed to the unsclerotized ovipositors of Cerambycinae, Lepturinae and Lamiinae (cf. e.g. Hubweber and Schmitt 2010: 40 Fig. 2).

#### Remarks.

*Sexual dimorphism*. The morphological differences between the sexes are largely confined to the anterior parts of the thoracic segments. The most noticeable difference lies in the appearance and structure of the pronotum. Although the outline is quite similar in both male and female. The pronotal disc of the male has an even, matte surface, while that of the female has a very irregular lustrous surface (Figure 2A). The male pronotum is mostly shallowly irrorate or punctate, with three deep depressions: two large lateral ones, elliptic in outline; and a smaller dot-like pit medially (Figure 1A). Conversely, the female pronotum is heavily and deeply sculptured, with two irregular smooth and raised areas in about the same position as the elliptic depressions of the male (Figure 2A).

Ventrally the male prosternum and proepimera have a relatively smooth surface, with only a shallow microstructure giving a matte appearance (Figure 1B), while the same parts in the female are strongly punctate and shiny (Figure 2B). The punctate prosternal process of the female is wider than the corresponding finely sculptured process of the male. Sexual dimorphism is also clearly expressed in the shape and structure of the mesosternum: where the female has a shiny mesial concavity anterior to the mesocoxae (Figure 2B), the male has a matte and finely punctate convexity with a low irregular, shiny median ridge (Figure 1B). The anterior border of the mesosternum, hidden beneath the prosternal process, is shallowly excavate in the female, but bluntly convex in the male. The bifurcate mesosternal process is quite prominent in the female, but much less so in the male.

As usual, the visible abdominal sternites are transversally more convex in the female than in the male (Figures 2A and 2B). Also, the pygidium is long and protruding in the female, but hardly visible in dorsal view in the male. The posterior border of the ultimate visible sternite is evenly rounded in the female, but weakly truncate in the male. Finally, unlike in most other Prioninae there is very little difference in the shape and length of the female and male antennae (Figures 1A and 1B).

*Male genitalia*. Bouyer (2012) in his original description of *Afraustraloderes rassei* pictured the male genitalia with lateral and dorsal views of the tegmen and ‘penis’ (median lobe), including the two sclerotized plates of the internal sac. The accompanying description was, however, very brief and limited in detail. A more comprehensive description is hereby given. Median lobe 4 mm long, strongly arcuate with apophyses (median struts, paired lamellae) rather short, constituting only c. 35% of total length of median lobe (Figure 4A); apophyses weakly sclerotized, becoming nearly transparent towards their truncate apices; ventral plate (ventral lobe of median lobe or “ventral edge of the median orifice” sensu Ehara 1954) very long with a sharply pointed apex, length surpassing the bilobed dorsal plate; ventral plate strongly sclerotized, dorsal plate much less so; median foramen not elongate; tegmen 4 mm long, strongly sclerotized (black almost throughout); parameres quite long, constituting one third of total tegmen length; apical brush with setae much shorter than parameres; ringed part gradually curved, not geniculate, arms converging (Figure 4B). The shape and structure of the anal tergite has in some studies proven to be of great diagnostic value (e.g. Adlbauer 1998). The anal tergite of the male *Afraustraloderes rassei* (Figure 4C) is black, about 1.5 times wider than long, moderately vaulted and with a truncate, but not emarginate, posterior border. The tergite is provided with a dense cover of black, short, stiff, very acute setae.

### Genus Pixodarus

Taxon classificationAnimaliaColeopteraCerambycidae

Fairmaire, 1887

#### Type species.

*Hoplideres nyassae* Bates, 1878

In their revision of the African and Madagascan Hopliderini (then referred to as subtribe Hopliderina), Quentin and Villiers (1972) cautioned strongly in their preamble about treating this group carefully: “due to the extreme variability of a number of characters” (l.c. p. 258). As examples of this extreme variability, they listed characters like the longitudinal furrow of the head, the length and number of spines on the antennae and antennomeres, the shape, sculpture and number of lateral spines on the pronotum, the spines and denticulation of the humeral and posthumeral margins of the elytra, and the pubescence on the ventral surface and tibiae.

In spite of these cautionary remarks, Quentin and Villiers (1972) described a new species of *Pixodarus*, *Pixodarus exasperatus*, based on very subtle characters. These included the raised parts of the pronotal vermiculations being glossy or not, variations in shades of reddish-brown to black in the colour of the elytra, as well as small differences in elytral length/width ratios.

*Pixodarus nyassae* is known from RSA and Mozambique northwards to Zimbabwe and Malawi, while *Pixodarus exasperatus* occurs in Tanzania, southern Kenya and the eastern part of the Democratic Republic of Congo (Quentin and Villiers 1972). During the course of this study, the authors have examined a number of specimens from RSA and Mozambique, as well as from Tanzania. We find great variation in all diagnostic characters mentioned both in the southern and the northern populations, and these variations strongly overlap. We therefore endorse the earlier proposal of Santos Ferreira (1980) to synonymize *Pixodarus exasperatus* with *Pixodarus nyassae*.

On the other hand, the female erroneously included in the description of *Afraustraloderes rassei* by Bouyer (2012) represents a new species of *Pixodarus* and, together with the recently obtained male, is described as a new species below.

### Pixodarus spiniscapus

Taxon classificationAnimaliaColeopteraCerambycidae

Bjørnstad & Grobbelaar sp. n.

http://zoobank.org/88E28DA9-EBA6-482D-BEC3-CEFCC6B4EF80

Figures 5 , 6 , 7

#### Type material.

Holotype ♂: SOUTH AFRICA: GAU, Florauna, Pretoria 25°41'20"S, 28°09'30"E, XII.1985, R.H. Watmough (SANC). Paratypes: 1♀: same data as holotype, XI.1989 (SANC); 1♂: same data as holotype, XII.1987 (ABPC); 1♀: RSA, GAU, Magaliesberg, SSW Hekpoort, 25°54'58"S, 27°35'51"E, 21-30.XI.2012, M. Shanahan (SANC).

#### Material excluded from type series due to insufficient data.

1♂: A69 [no further data] (SANC).

#### Additional records (iSpot).

1♂: RSA, GAU, Roodepoort, Walter Sisulu National Botanical Garden (26°05'21"S, 27°50'40"E), 05 Nov 2012, A Hankey, http://www.webcitation.org/6ZkmxK0on; 1♀: RSA, GAU, Randburg, Curro Aurora Private School, school grounds (26°04'48"S, 27°56'06"E), 30 Oct 2014, A Hankey, http://www.webcitation.org/6ZknFLtcm; 1♂: RSA, MPU, Presidentsrus (25°45'33"S, 29°19'05"E), 31 Oct 2014, R Bate, light trap, http://www.webcitation.org/6ZknAyhMq; 1♀, ditto, but 14 Nov 2014, http://www.webcitation.org/6ZknGm829.

#### Description.

*Size*. Male: 31.5–33.7 mm long, 12.5–13.4 mm wide (maximum width at metacoxae); female: 33.9-34.0 mm long, 13.0-13.7 mm wide (maximum width at metacoxae).

*Head*. Mandibles black, short and stout with deeply punctate base and strongly hooked apex; maxillary palpi tetramerous, labial palpi trimerous, basal segment of maxillary palpi extremely short and appearing 3-segmented; shape and size of the two types of palpi very similar, both shiny and brown with long, stiff, yellow setae, terminal segments hyaline in apical third and abruptly truncate; frons short, deeply concave, bordered by lateral ridges formed as continuation of mandibular bases; antennal tubercles prominent, separated by a narrow depression which continues well past the eyes posteriorly, then disappears gradually on vertex; yellowish to orange arcuate to curled bristles present on most surface areas, particularly well-developed along above mentioned depression; entire fronto-dorsal surface heavily sculpted with irregular irrorations and foveolations; eyes finely facetted, sinuately emarginate resulting in smaller dorsal eye lobe and larger suborbicular ventral lobe, emargination itself densely pubescent; genae very short.

*Antenna*. Scape almost wedge-shaped, gradually widening distally from narrow base, slightly compressed, with marked apical spine on proximal side; pedicel very short (< 1/5 length of scape); antennomere 4 noticeably shorter than 3 and 5 in male, not in female; antennomeres 3 and 5–10 of subequal length in male; last antennomere longest by far, more than 1.5 times length of preceding; antennomeres 3–9 + 11 of subequal length in female, only antennomere 10 slightly shorter; antennomeres 1–3 punctate in both male and female, segment 4 transitional and last 7 antennomeres only very finely micropunctate; antennomeres 2–4 practically terete, from segment 5 onwards increasingly flattened and with weak lateral tooth apically; apical segment sharply carinate; antenna reaching approximately 4/5 of elytral length or slightly longer in male, only about ½ or slightly longer in female.

*Pronotum*. Distinctly transverse, length/width ratio of pronotal disc (lateral spines excluded) approximately 0.6; anterior margin straight to shallowly concave, posterior margin slightly convex medially; three stout spines present laterally on either side, two straight spines at edge of anterior margin and at posterior corner respectively, one strongly curved spine between previous two and pointing posteriorly; few irregular small teeth between first and second spine; pronotal disc irregularly folded and foveate to shallowly vermiculate, elevated parts shiny; sparse yellowish- to rusty brown and somewhat curly pubescence covering much of surface, particularly dense along anterior margin.

*Scutellum*. Shield-like, pubescent and finely punctate.

*Elytron*. Dark brown, and rather elongate, ratio elytral length:combined elytral width at metacoxae varying between 1.9 and 2.3; surface distinctly costate, with three costae normally visible at least in part; humeri rounded and weakly marked; posthumeral lateral margin bent outwards and upwards, forming a miniature “gutter”; posterior sutural corner sharply angled, or exhibiting small tooth, otherwise lateral margin evenly rounded; shallow vermiculations in subscutellar part, gradually becoming finely punctate laterally and posteriorly; basal parts sparsely covered with short yellowish pubescence, gradually becoming less conspicuous posteriorly; greatest elytral width in female about half way down the elytron, distinctly wider than in male.

*Legs*. Profemora with a somewhat scabrid dorsal surface, ventral face punctate; meso- and metafemora smooth and lustrous dorsally; tibiae straight with short rusty brown pubescence, all widening towards the apex, with two short spurs on proximal side and blunt tooth on distal side; all tarsomeres on all legs of subequal length.

*Ventral surface*. Gula strongly concave and densely punctate in anterior part, transversely undulate in posterior part; prosternum pubescent, distinctly convex with anterior border strongly thickened; prosternal process ligulate with apex bent dorsally; procoxal cavities open; mesosternum punctate and pubescent, very short; mesosternal process short with median furrow, bifurcate apically; metasternum strongly convex, pubescent, finely punctate and glossy; all five visible abdominal sternites of subequal length, finely punctate, pubescent and glossy; last visible sternite with rounded to weakly truncate posterior border in female, weakly concave in male; epipleura well developed in correspondence with dorsal gutter-like extension of elytral margin.

*Male genitalia*. Median lobe (sensu Ehara 1954, Hubweber and Schmitt 2010; otherwise commonly referred to as just “aedeagus” or “penis”, e.g. Bouyer 2012) with heavily sclerotized acuminate ventral edge of median orifice (= ventral plate sensu Lin and Li 2012, but usually referred to as ‘ventral lobe’); dorsal edge of median orifice (dorsal plate/‘dorsal lobe’) rounded with emarginate apex, only slightly shorter than ventral edge, and distinctly less sclerotized; basal apophyses (sensu Hubweber and Schmitt 2010; in Ehara 1954 as “median struts”) long and strap-shaped, constituting more than 60% of total length of median lobe; median foramen not elongate; tegmen reddish-brown to brown, indicating medium sclerotization (Hubweber and Schmitt 2006); parameres (lateral lobes) long and slender and widening slightly apically, with brush of setae, shorter than the parameres; pouch-like appendage on internal side at base of each paramere; tegminal ring with converging geniculated arms.

#### Etymology.

The name ‘*spiniscapus*’ refers to the prominent apical spine present on the first antennomere or scape.

#### Remarks.

*Pixodarus spiniscapus* sp. n. differs from *Pixodarus nyassae* (Bates, 1878) (syn. *Pixodarus exasperatus* Quentin and Villiers, 1972) in several diagnostic characters. Unlike *Pixodarus nyassae*, *Pixodarus spiniscapus* has costate elytra and these are also more elongate than in *Pixodarus nyassae*. Its ratio of elytral length: combined width at humeri varies from 1.9 to 2.3, while it ranges between 1.7 and 1.9 in *Pixodarus nyassae*. The first antennomere, in particular, has a distinct apical spine in *Pixodarus spiniscapus*, while it is unarmed in *Pixodarus nyassae*. Furthermore, in *Pixodarus spiniscapus* most of the pronotal disc and elytral surfaces have a yellowish pubescence, which is lacking in *Pixodarus nyassae*. The pronotum in *Pixodarus spiniscapus* has three lateral spines on either side, while there are usually five in *Pixodarus nyassae*. There are also significant genitalic diagnostics. While the median lobe of *Pixodarus spiniscapus* is very similar to that of *Pixodarus nyassae*, the ventral edge of the median orifice (ventral plate) in particular has an acuminate apex longer than that of *Pixodarus nyassae*. In the terminology of Ehara (1954: 65), that of *Pixodarus spiniscapus* would classify as ‘sharply pointed’ (l.c. Fig. 4), while that of *Pixodarus nyassae* looks more like ‘strongly projected’ (l.c. Fig. 7). The dorsal edge of the median orifice (‘dorsal lobe’) in *Pixodarus spiniscapus* has a more deeply emarginate apex than in *Pixodarus nyassae*. The tegmen has parameres with setae confined to the widened apical part in *Pixodarus spiniscapus*, but in *Pixodarus nyassae* the apical part is not noticeably wider than the basal part, and the setae cover a larger part of the surface.

**Figure 5.**
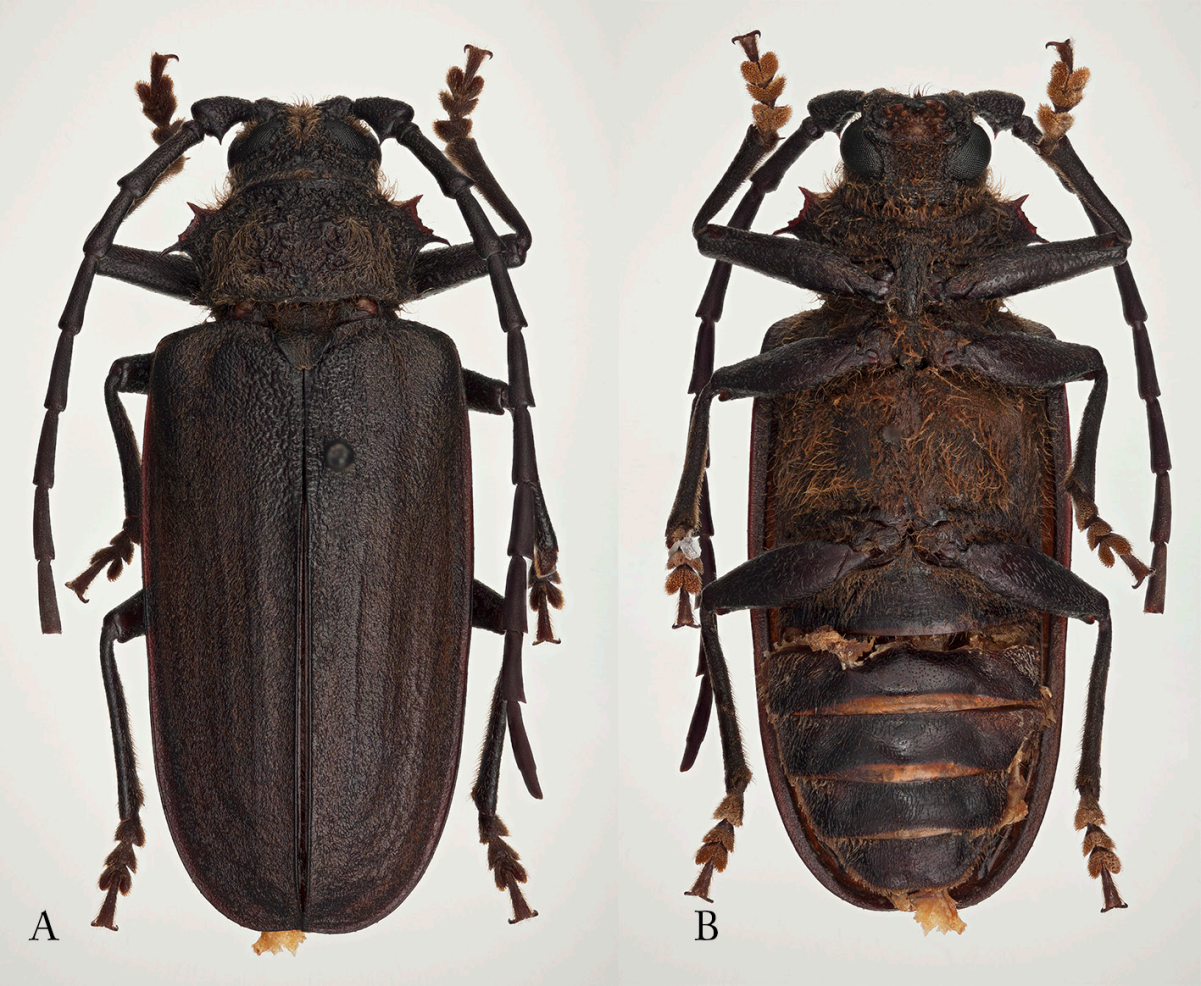
*Pixodarus spiniscapus* sp. n. holotype ♂, 31.5 mm **A** dorsal habitus **B** ventral habitus (Photos: Karsten Sund).

**Figure 6.**
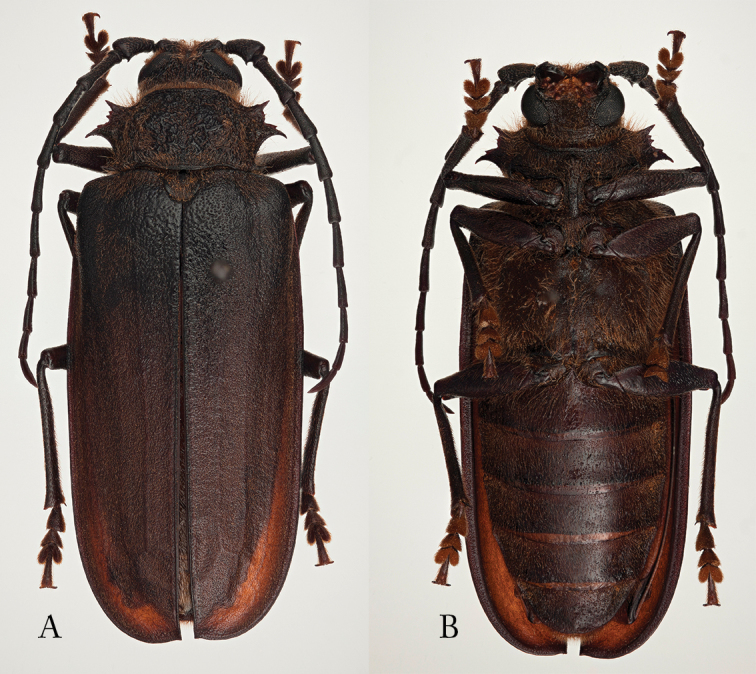
*Pixodarus spiniscapus* sp. n.: paratype ♀, 33 mm **A** dorsal habitus **B** ventral habitus (Photos: Karsten Sund).

**Figure 7.**
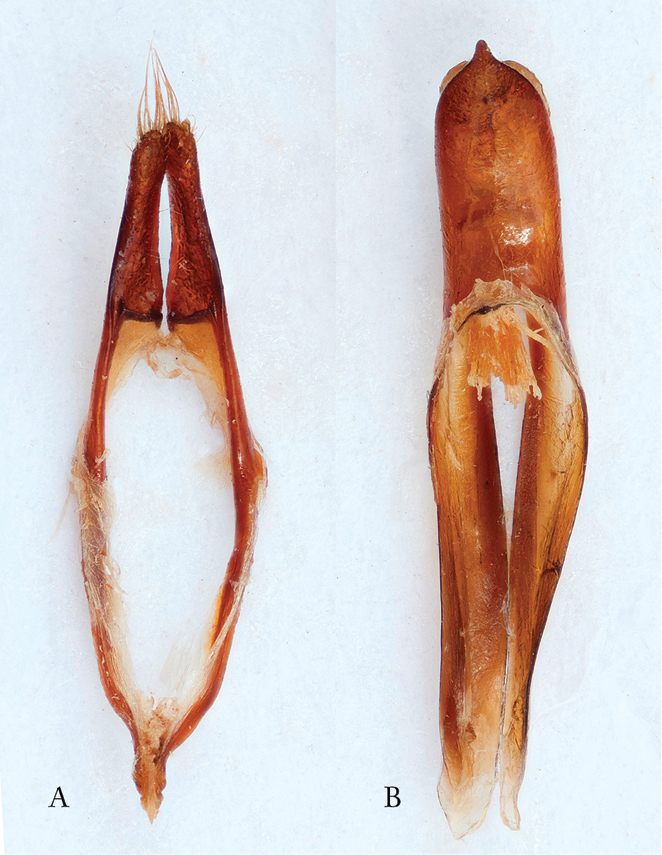
*Pixodarus spiniscapus* sp. n. male genitalia **A** tegmen, ventral view **B** median lobe (Photos: Anders Bjørnstad and Karsten Sund).

## Discussion

This study shows that the two sexes originally described as belonging to the same species, *Afraustraloderes rassei*, actually represent two very different species. The unique characteristics of the genus *Afraustraloderes* are also highlighted, revealing the unlikelihood of it belonging to the tribe Hopliderini. According to the analysis undertaken by Quentin and Villiers (1972), this tribe is a coherent systematic group with no obvious outliers. The main reason used by Bouyer (2012) for including *Afraustraloderes* in the Hopliderini was its perceived association with what is now known as the female of a true *Pixodarus*. However, *Afraustraloderes* is something that cannot be compared closely with anything else, at least on the African continent, and may even constitute a tribe of its own. A phylogenetic study, including molecular analyses, is thus required to resolve the systematic position of this genus.

The habitat and ecology of *Afraustraloderes rassei* are also unusual for a prionine – both adults and larvae have so far only been found in fynbos vegetation (Figure 7), with a distribution range apparently restricted to the Fynbos Biome. Most specimens have been collected on the slopes of the Langkloof-Tsitsikamma mountain range in the Joubertina area of the Eastern Cape Province, at an altitude of approximately 650 m. The holotype described by Bouyer (2012) reportedly originates from Matroosberg, which is located at the entrance of the Hex River Valley, near Worcester in the Western Cape Province. The two localities are approximately 400 km apart, but both are well within the Fynbos Biome (Mucina and Rutherford 2006).

**Figure 8.**
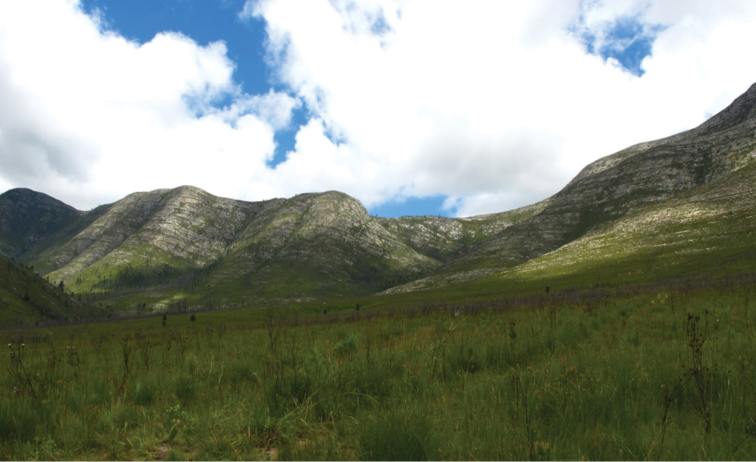
Typical habitat of *Afraustraloderes rassei*, with fynbos vegetation regrowth after fire on the slopes of the Langkloof mountains near Joubertina (Photo: Lynette Clennell).

Among the fynbos vegetation, adults and larvae of *Afraustraloderes rassei* were found on or inside trunks, branches and upper roots of dead *Protea* and *Leucadendron* species (Proteaceae) and also *Hakea sericea* Schrad. and J.C.Wendl., which is an alien invasive plant in the same family, introduced into South Africa from southeastern Australia in the 19^th^ century (Rebelo 2001). All adults were found during the daytime, perched on branches or crawling at the base of dead plants. They exhibit very sluggish movements and when disturbed assume a carabid-like defence position, raising their abdomen above the ground and pointing the pygidium in the direction of the would-be predator (Figure 8). They raise the abdomen, then continuously lower and raise it in a pump-like movement, when disturbed. It is possible that they mimic the behaviour of beetles of the genus *Termophilum* Basilewsky, 1950 (Carabidae: Harpalinae), which are quite common in the area and which spray powerful chemical concoctions, based on formic acid, towards potential predators as a deterrent (Scholtz and Holm 1985). It is not yet known whether adults are also active at night, but light trapping undertaken in the area on several occasions during the period 2010–2014 failed to provide any evidence that the species may be nocturnal and/or attracted to light.

**Figure 9.**
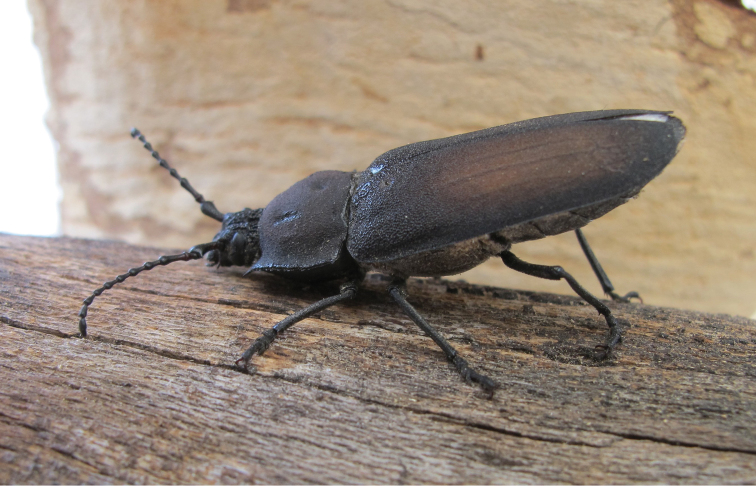
*Afraustraloderes rassei* ♂ specimen in its natural habitat assuming “defensive position”, Joubertina 25 Dec 2010 (Photo: Lynette Clennell).

Larval stages of *Afraustraloderes rassei* were first collected in December 2010, but were small, < 15 mm long, and did not survive to the next season in captivity. By December 2013, larvae observed in their natural habitat had reached an average size of 30–50 mm and were able to survive in captivity, with a few eventually pupating and emerging as adults in December 2014–January 2015. Others are currently completing their life cycle in the laboratory. As the fynbos area where larvae were monitored had been burned by a veld fire in the winter of 2009, it would appear that larvae may take about 6–7 years to complete their development to imago. Thus, it seems likely that this reflects the cycle of natural fire burning in the fynbos vegetation of this region (Van Wilgen et al. 1992). This will provide a regular, new supply of dead plants at a rate comparable to that of the beetle life cycle.

Unfortunately, very little is known about the habitat and biology of *Pixodarus spiniscapus*, as all known specimens are accompanied by essential data only. It is most likely that the specimens collected by RH Watmough in the Florauna suburb of Pretoria were attracted to the light at night, as this is the typical behavior shown by the other known species of the genus *Pixodarus* (RP, pers. observ.), as also confirmed by the iSpot observations. The predominant vegetation of the Magaliesberg slopes, where the bulk of the type specimens of *Pixodarus spiniscapus* were collected, is of the Gold Reef Mountain Bushveld type (Mucina and Rutherford 2006). Specimens recorded on iSpot occur in Rand Highveld Grassland which has an intrusion of Loskop Mountain Bushveld along the river course about 13 km to the north (Presidentsrus which lies alongside the river); Gold Reef Mountain Bushveld (Walter Sisulu National Botanical Garden); and Egoli Granite Grassland (Randburg, grounds of the Curro Aurora Private School) (Mucina and Rutherford 2006). The specimen erroneously included as “allotype female” in the description of *Afraustraloderes rassei* by Bouyer (2012), was reportedly from the Oribi Gorge area of KwaZulu-Natal. However, one of the authors (RP) has collected cerambycids intensively in the Oribi Gorge Nature Reserve over the past 15 years, including by light trapping at night, and has never encountered this species. Given that the specimen was acquired at an unspecified fair and carries no collector’s name, it is possible that the locality reported in the data label may be erroneous.

## Supplementary Material

XML Treatment for Afraustraloderes rassei

XML Treatment for Pixodarus

XML Treatment for Pixodarus spiniscapus
